# Effects of artepillin C on model membranes displaying liquid immiscibility

**DOI:** 10.1590/1414-431X20198281

**Published:** 2019-03-25

**Authors:** W.M. Pazin, N. Vilanova, I.K. Voets, A.E.E. Soares, A.S. Ito

**Affiliations:** 1 Departmento de Física, Faculdade de Filosofia, Ciências e Letras de Ribeirão Preto, Universidade de São Paulo, Ribeirão Preto, SP, Brasil; 2 Departmento de Física, Faculdade de Ciências e Tecnologia, Universidade do Estado de São Paulo, Presidente Prudente, SP, Brasil; 3 Macromolecular and Organic Chemistry, Physical Chemistry & Institute for Complex Molecular Systems, Eindhoven University of Technology, Eindhoven, The Netherlands; 4 Dutch Polymer Institute (DPI), Eindhoven, The Netherlands; 5 Departamento de Genética, Faculdade de Medicina de Ribeirão Preto, Universidade de São Paulo, Ribeirão Preto, SP, Brasil

**Keywords:** Artepillin C, Green propolis, Model membranes, Giant unilamellar vesicles, SAXS, DSC

## Abstract

It has been hypothesized that the therapeutic effects of artepillin C, a natural compound derived from Brazilian green propolis, are likely related to its partition in the lipid bilayer component of biological membranes. To test this hypothesis, we investigated the effects of the major compound of green propolis, artepillin C, on model membranes (small and giant unilamelar vesicles) composed of ternary lipid mixtures containing cholesterol, which display liquid-ordered (l_o_) and liquid-disordered (l_d_) phase coexistence. Specifically, we explored potential changes in relevant membrane parameters upon addition of artepillin C presenting both neutral and deprotonated states by means of small angle X-ray scattering (SAXS), differential scanning calorimetry (DSC), and confocal and multiphoton excitation fluorescence microscopy. Thermotropic analysis obtained from DSC experiments indicated a loss in the lipid cooperativity of l_o_ phase at equilibrium conditions, while at similar conditions spontaneous formation of unilamellar vesicles from SAXS experiments showed that deprotonated artepillin C preferentially located at the surface of the membrane. Time-resolved experiments using fluorescence microscopy showed that at doses above 100 µM, artepillin C in its neutral state interacted with both liquid-ordered and liquid-disordered phases, inducing curvature stress and promoting dehydration at the membrane interface.

## Introduction

Depending on the lateral organization of the lipid bilayer and the nature of the lipid's polar head group, bioactive compounds can interact with biological membranes and display a range of therapeutic effects by altering their structural and dynamical properties, leading to cell death (1–4). Natural compounds found in Brazilian biomes have been a target of several studies due to the variety of species spread across the country and their large spectrum of biological activities. Due to their particular chemical structures, these compounds are able to interact directly with lipid bilayers without the presence of specific membrane receptors. Examples of these compounds are antimicrobial peptides and secondary metabolites extracted from plants, such as phenolic compounds, which have been the subject of many studies (1,5,6).

Propolis is a product originated from the mixture of beeswax and resinous compounds that bees selectively collect from the vegetation. Propolis contains high amounts of bioactive secondary metabolites necessary to guarantee asepsis for beehives and protect them against intruders (7–9). Among all the propolis types, Brazilian green propolis, collected by the bee species *Apis mellifera*, has been extensively investigated worldwide, due to the presence of artepillin C (3,5-diprenyl-4-hydroxycinnamic acid), a phenolic acid derivative compound, which presents antioxidant, anti-inflammatory, and antitumor properties (10–12). This molecule has two prenylated groups bound to a phenyl group, which enhances its hydrophobicity (see Supplementary Figure S1).

It is broadly accepted that lipid bilayers are essential structures for the function and integrity of cells. Simple model membranes formed by distinct lipids have been investigated for decades to obtain important biophysical parameters related to these peculiar supramolecular structures. Taking into account the taxonomic classification of the studied cells, variations of the polar head group and/or the hydrophobic tail of the lipids result in relevant model systems that mimic cell membranes (2 [Bibr B03],^13^–^15^). It has been shown that, depending on their composition, lipid membranes may show specific lateral structures, characterized currently as membrane domains, which can associate distinct lipids and proteins (16,17). It is hypothesized that these structures may play an important role in many biological processes, such as protein trafficking and signaling processes. Model membranes formed of ternary lipid mixtures, particularly composed of cholesterol, have become a popular system to model the lipid component of biological membranes. A general feature of these lipid mixtures is that they display liquid immiscibility (18–22).

In a previous study, we tested the interaction of artepillin C with a compositionally simple model membrane formed by 1,2-dimyristoyl-sn-glycero-3-phosphocholine (DMPC) using several experimental techniques and molecular dynamic simulations (13). In such study, we found that, in an aqueous lipid dispersion, the compound preferentially partitions to the lipid membrane disturbing the lateral organization and thermotropic behavior of DMPC bilayers. Recently, we have evaluated the interaction of artepillin in different protonation states with model membranes formed by 1,2-dipalmitoyl-sn-glycero-3-phosphocholine (DPPC), showing that the neutral state of the compound has a higher affinity for lipophilic environment compared to deprotonated species (23). When in ultra-pure Milli-Q water, artepillin C presents both neutral and deprotonated populations (W.M. Pazin, A.S. Ito, unpublished results), since the measured pH of this particular system is about 4.9 (24). In order to evaluate the effect of this compound on lipid membranes showing higher compositional complexity, we examined the interaction of artepillin C on membrane systems composed of mixtures of DPPC, 1,2-dioleoyl-sn-glycero-3-phosphocholine (DOPC), and cholesterol suspended in ultra-pure Milli-Q water, which displays coexistence of liquid-ordered (l_o_) and liquid-disordered (l_d_) phases (25–27). The coexistence of these two phases is thought to mimic distinct membrane sub-compartments relevant to describe biological membranes (28). We used as main experimental tools confocal and two photon excitation fluorescence microscopy, together with differential scanning calorimetry (DSC) and small angle X-ray scattering (SAXS). While the former technique allows spatiotemporal analyses (morphology, local curvature, and hydration) of the specific phases existing in the lipid bilayer, the last two techniques, respectively, allowed inquiring effects caused by artepillin C in the thermotropic lipid phase transition and changes in the structural properties of the model membranes at equilibrium conditions.

## Material and Methods

### Chemicals

DOPC, DPPC, and cholesterol were purchased from Avanti Polar Lipids (USA). Artepillin C, isolated and purified from Brazilian green propolis, was purchased from Wako, Japan. 1,19-dioctadecyl-3,3,39,39-tetramethylindocarbocyanine perchlorate (DiI-C_18_) was from Molecular Probes (USA) and 6-dodecanoyl-2-dimethylamino naphthalene (laurdan) was obtained from Invitrogen (Denmark). All the reagents were used without further purification. Lipid suspensions were prepared by using filtrated Milli-Q water (18.2 MΩ^.^cm).

### Preparation of model membranes

Large unilamellar vesicles (LUVs) and multilamellar vesicles (MLVs) were prepared from stock solutions of DOPC, DPPC, and cholesterol in chloroform at 40 mM with a final lipid molar ratio of 23:47:30, respectively. As reported in the literature (19 [Bibr B20],^27^,^29^), this particular composition exhibited liquid-ordered and liquid-disordered phase coexistence from 20°C to up to 33°C. Vesicles (LUVs and MLVs) containing artepillin C were prepared by premixing the lipid organic solution with appropriate aliquots from a stock solution of this compound (20 mM) in methanol (MeOH). As a first step, the organic mixture was dried by carefully forming films of the sample on the wall of glass test tubes under a N_2_ flow. To eliminate remaining traces of organic solvent, the samples were placed in a desiccator under reduced pressure for at least 1 h. MLVs were prepared at 45°C by adding Milli-Q water onto the dried samples and sequentially mixed for periods of 2 min using a vortex until the films disappeared from the glass tube walls. LUVs were prepared by extruding MLVs through a polycarbonate membranes containing 0.1 µm pores (Whatman, Sigma Aldrich, USA) at least 21 times, as described elsewhere (14,30).

Giant unilamellar vesicles (GUVs) were prepared by the electroformation method developed by Angelova and Dimitrov (31) in a homemade temperature-controlled chamber as described previously (Husen et al., 2012). Briefly, aliquots of 4 µL of a solution containing DOPC/DPPC/cholesterol (23:47:30 molar ratio; 0.2 mg/mL) in chloroform, doped with either 0.5 mol% of DiIC18 or 2 mol% of laurdan, were spread on each platinum electrode. The chamber was then placed under vacuum for at least 1 h to remove any remaining traces of organic solvent. The electroformation was carried out by covering the electrodes with solutions of sucrose 200 mOsM (0.4 mL final volume) at temperatures above the lipid phase transition (45°C) and applying a low-frequency alternating field (sinusoidal wave function with a frequency of 10 Hz and amplitude of 1 V) for 120 min using a function generator (Digimess FG 100). The AC field was turned off, and the GUVs were harvested from the chamber.

### Small angle X-ray scattering (SAXS)

SAXS measurements were performed on a SAXSLAB GANESHA 300 XL SAXS system (Denmark) equipped with a GeniX 3D Cu ultra low divergence micro focus sealed tube source (France) that produced X-rays with a wavelength of *λ=*1.54 Å. A sample-to-detector distance of 713 mm was used to access a *q*-range of 0.15 ≤ *q* ≤ 4.47 nm^−1^ with *q=*4*π*/*λ*(sin *θ*), where *2θ* was the angle between the incident X-ray beam and the detector measuring the scattered intensity. Extruded lipid suspensions of DOPC/DPPC/cholesterol (23:47:30 molar ratio; 40 mM) with and without 10 mol% of artepillin C were placed in 2-mm quartz capillaries (Germany). The sample temperature was kept at 30°C with the aid of a Julabo temperature controller (Germany). The acquisition time of the SAXS data was 6 hours for each sample, and the background signal (scattering of a capillary filled with water) was subtracted from the obtained profiles. The experimental SAXS diagrams were fitted by using the Global Analysis program (GAP) version 1.3, provided by Dr. Georg Pabst of the Austrian Academy of Sciences – Graz. Herewith, we obtained the electron density profile of the polar head group, of the acyl chain regions of the lipid bilayers, and of the fraction of the resulting unilamellar vesicles (32,33) by using the function:

(Eq. 1) $$l(q)=(1-N_{UV})\frac{S(q)|F(q){|}^{2}}{q^{2}}+N_{UV}\frac{|F(q){|}^{2}}{q^{2}},$$

where *NUV* is the fraction number of positionally non-correlated particles (i.e., unilamellar vesicles), *S(q)* is the structure factor (inter-particle interaction), and *F(q)* is the form factor, which gives the electron density profile. From the parameters that describe the head group regions, it was possible to calculate the thickness of the membrane (*d*B) through the following equation (34):

(Eq. 2) $$d_{B}=2(Z_{H}+2\sigma_{H}),$$

where *z_H_* is the headgroup position measured from the center of the bilayer, and *σ_H_* is the width of the Gaussian of the electron-dense distribution over the headgroup region.

### Differential scanning calorimetry (DSC)

Experiments were carried out on a VP-Capillary DSC calorimeter from Microcal (USA). Degassed aqueous suspensions of MLVs (10 mM) composed of DOPC/DPPC/cholesterol 23:47:30 mol with (1, 5, and 10 mol%) or without artepillin C were placed in the calorimeter. The scan rate was 0.5°C/min for all experiments. The Microcal Origin software, provided by Microcal, was used to subtract the baseline and analyze the data. Each sample was scanned at least seven times. The experiments were performed in triplicates.

### Morphologic analysis by confocal fluorescence microscopy

GUVs were observed in an inverted confocal microscope (Zeiss LSM 510 META, Germany) using a 60× water immersion objective, NA 1.4. For the DiC18 experiments, we used an excitation wavelength of 543 nm (HeNe laser source). The excitation light was reflected to the sample through a dichroic mirror HFT 488/543/633 and the emission collected using a NFT 545 filter placed at the front of a photomultiplier (PMT) detector. Aliquots of 50 µL of DOPC/DPPC/cholesterol 23:47:30 mol GUVs suspended in glucose 200 mOsM and labelled with DiIC_18_ were transferred to each of an eight-well plastic chamber (Ibidi, Germany) containing 0.3 mL of iso-osmolar glucose solution. The density difference between the interior and exterior of the GUVs caused by the two sugar solutions promotes the vesicles to sink to the bottom of the chamber facilitating their observation in an inverted microscope. Proper aliquots of artepillin C 20 mM in methanol (or pure methanol 1% (v/v), used as negative control) were injected into the chamber, and time-series scans were initiated to monitor GUVs up to 90 min. The experiments were performed at least in triplicate. After the injections, a minimum of 20 vesicles were monitored as a function of time for each experiment at 25°C. The images were analyzed by the software ImageJ.

### Laurdan generalized polarization (GP) images

Laurdan-GP denotes the position of the laurdan emission spectrum, which depends on the extent of water dipolar relaxation processes that occurs in the probe's environment (23). Specifically, the emission spectrum of laurdan is sensitive to the amount and dynamics of water molecules existing at the glycerol backbone region of the lipids, which are highly dependent on the lipid packing (35). In a disordered lipid phase, the rotational dynamics of water molecules near the transition moment of laurdan is in the order of the probe's lifetime, decreasing the energy of the excited state and causing a red shift of the fluorescence emission. In contrast, in membrane regions where a more ordered packing exists (liquid ordered or gel), the rotational dynamics of water is much lower than the probe lifetime and the energy of the excited state is unaffected, i.e. the emission is blue-shifted with respect to the liquid disordered phase. These changes are reflected in the generalized polarization function (36) defined as:

(Eq. 3) $$GP=\frac{I_{440}-I_{490}}{I_{440}+I_{490}}$$

where *I*_440_ and *I*_490_ correspond to the position of the laurdan spectrum at 440 and 490 nm using a given excitation wavelength.

In order to obtain GP images, we used a custom-built multiphoton excitation microscope constructed on an Olympus IX70 microscope. A femtosecond Ti:Sa laser (Broadband Mai Tai XF-W2S with 10W Millennia pump laser, tunable excitation range 710–980 nm, Spectra Physics, USA) tuned at 780 nm was used as an excitation wavelength source. The objective used in the experiments was a 60× water immersion, NA of 1.2. The fluorescence signal was collected in two separate detectors (Hamamatsu H7422P-40) by splitting the fluorescence with a dichroic mirror above and below 475 nm. Each detector contained an additional bandpass filter (438±12 nm and 494±10 nm) allowing to simultaneously acquire intensity images corresponding to the blue and red sides of the laurdan spectrum (i.e., *I*_440_ and *I*_490_, respectively; see equation 3), which are necessary to compute the GP images. The GP images were calibrated using a correcting G factor as reported elsewhere (37) using a laurdan-GP standard in DMSO. All laurdan-GP images were computed using SIMFCS program, developed by the Laboratory for Fluorescence Dynamics, University of California at Irvine, USA. The time series obtained for the GP images of DOPC/DPPC/cholesterol (23:47:30 molar ratio) GUVs (labelled with laurdan) upon interaction with artepillin C were performed using the same experimental strategy indicated above. Images of GUVs were acquired in the equatorial region of the GUVs to avoid the probe's photoselection effect (25).

## Results

### Differential scanning calorimetry

**Figure 1.**
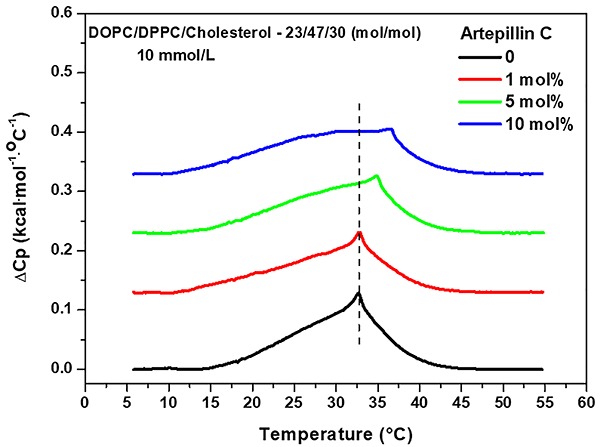
Differential scanning calorimetry thermotropic curves of lipid suspension (10 mM) of multilamellar vesicles formed of DOPC/DPPC/cholesterol (23:47:30 molar ratio), in the absence and presence of 1, 5, and 10 mol% of artepillin C. Temperature range was from 5 to 55°C.

Thermograms of the mixture DOPC:DPPC:cholesterol (23:47:30 molar ratio) obtained by DSC were examined in the range of temperatures from 5 to 55°C, which corresponded to the temperature region where the pre- and main phase transition of pure DPPC membranes (undergoing from gel -L_β'_- to a fluid phase -L_α_- at 41°C) is observed (14). The broad thermal transition measured for the lipid suspension in the absence of artepillin C (black curve in Figure 1) in this temperature range, results mainly from the presence of cholesterol embedded in DPPC enriched domains (L_o_ domains), resulting in a substantial loss of cooperativity observed for pure DPPC (38). Specifically, cholesterol affects the DPPC thermal phase transition by increasing its fluidity, a situation that is reflected by a broad endotherm with a lower main transition temperature (Tm=32.6°C). Such effects were reported by Fritzsching et al. (26) for the DOPC/DPPC/cholesterol system, suggesting that cholesterol, at high concentrations, preferentially locates in DPPC-enriched domains present in the mixture, hence vanishing its phase transition. By adding 1 mol% of artepillin C into the lipid suspension, the thermotropic properties are barely changed (red curve in Figure 1), maintaining both transition temperature and enthalpy change as for the vesicles without artepillin C. Upon an increase of artepillin C between 5 to 10 mol%, a shift of T_m_ to higher values is observed, from 34.9 to 36.5°C, respectively (green and blue curves in Figure 1). Moreover, the increase in the difference between the lower and upper boundaries of the transition peaks upon artepillin C addition suggested a loss in the cooperativity of the transition. The width at half height of the peak (T_1/2_) was calculated in order to obtain information about the cooperativity of the phase transition, which described the number of lipids involved in the transition, i.e., the so-called cooperative unit (CU). This parameter is calculated by the ratio of ΔH_vH_/ΔH_c_, where ΔH_c_ is the enthalpy of the transition (cal/mol) and ΔH_vH_ is the van't Hoff enthalpy. The van't Hoff enthalpy can be calculated by an approximated expression, which depends on T_m_ and T_1/2_ according to:

(Eq. 4) $$∆H_{VH}≅\frac{4RT_{m}^{2}}{T_{1/2}}$$

where *R* is the gas constant. CU, for a purely cooperative first order transition tends to infinity, while for a non-cooperativity transition, CU reaches zero. Table 1 reports both ΔH_c_ and ΔH_VH_, as well as the CU for all studied systems. Despite observations regarding peak alterations, the enthalpy change of the transition was independent of artepillin C concentrations. However, ΔH_VH_ diminished according to artepillin C concentration, and, by calculating CU, the mean value indicated that cooperativity of the transition of the lipid mixture tended to decrease for the interaction of this compound at 5 and 10 mol%. Thermal analyses of artepillin C were not investigated in the range of DOPC phase transition (–20°C), due to equipment restriction for low temperature acquisitions (below 0°C).

**Table 1.** Thermodynamic data obtained from differential scanning calorimetry measurements of DOPC/DPPC/cholesterol (23:47:30 molar ratio), 10 mM, in the absence and presence of artepillin C-inserted vesicles (1, 5, and 10 mol%).

Artepillin C (mol%)	DOPC/DPPC/cholesterol (23:47:30 molar ratio - 10 mmol/L)			
	T_m_ (°C)	ΔH_C_ (kcal/mol)	ΔH_VH_ (kcal/mol)	CU
0	32.6	1.4±0.3	75.8	54±11
1	32.6	1.2±0.4	68.1	56±18
5	34.9	1.3±0.3	54.1	42±9
10	36.5	1.4±0.3	41.2	29±6

Data are reported as mean±SD. T_m_: transition temperature; ΔH_C_: transition enthalpy; ΔH_VH_: van't Hoff enthalpy; CU: cooperative unit.

### SAXS analysis

**Figure 2.**
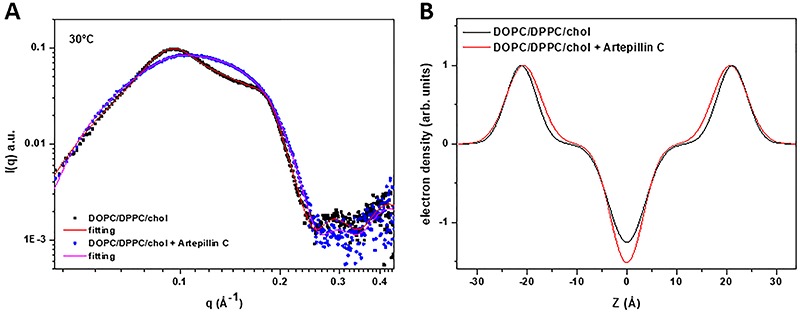
Small angle X-ray scattering (SAXS) diagrams (**A**) and electron density profiles (**B**) of extruded lipid vesicles formed of DOPC/DPPC/cholesterol (23:47:30 molar ratio), 40 mM, in the absence and presence of 10 mol% of artepillin C, obtained at 30°C. Electron density profiles were obtained by fitting the SAXS curves with the aid of the Global Analysis Program software (version 1.3).

SAXS was applied to get information regarding bilayer thickness once thermal experiments have shown that artepillin C caused loss in the lipid cooperativity, which could be a consequence of changes in the lipid packing of the bilayers. The SAXS pattern of extruded vesicles formed by DOPC/DPPC/cholesterol (23:47:30 molar ratio) in the absence of artepillin C showed 3 to 4 orders of broad quasi-Bragg diffraction peaks in the presented diagram, with prominent one around 0.1 Å^-1^ and a shoulder around 0.2 Å^-1^ (Figure 2A), indicative of the presence of multilamellar vesicles despite the extrusion procedure (13,30). Even though the improvement of LUVs formation could be achieved by additional procedures, for instance addition of a small percentage of negatively charged lipids to the suspension (39), the SAXS pattern of the vesicles with artepillin C indicated that the insertion of the bioactive compound in the bilayer also favored the formation of unilamellar vesicles, as the quasi-Bragg peaks was absent. From the fitting analysis using equation (1), the percentages of unilamellar vesicles (LUVs) in the absence and in the presence of artepillin C were calculated as 52 and 87%, respectively, as reported in Table 2. The electrostatic repulsion introduced by the negative charge of deprotonated species of artepillin C, located at the water/lipid interface, would favor the spontaneous formation of unilamellar vesicles, as previously reported for with DMPC bilayers (13). The *d*_B_ value calculated through equation (2), points out that the presence of artepillin C slightly decreased the average thickness of the bilayers, going from 50.2±0.4 to 48.0±1.0 Å, reinforcing the idea that the decrease in the CU as shown by DSC experiments could be a consequence of a slight increase in the fluidity of the lipid bilayer caused by a loss in the lipid packing, probably affected by the presence of the protonated species of artepillin C even more embedded in the lipid membrane compared to deprotonated species, in agreement with previous studies (23). Therefore, the d-spacing increased upon artepillin C interaction from 65.8±0.1 to 71.4±0.5 Å. This parameter is the sum of *d*_B_ plus the space between the bilayers into the remaining oligolamellar vesicles in the system, and thus its increase confirmed the electrostatic repulsion caused by artepillin C, pushing out the bilayers from each other, resulting in a larger overall thickness.

**Table 2.** Structural parameters obtained by fitting the small angle X-ray scattering profiles of extruded vesicles formed of DOPC/DPPC/cholesterol (23:47:30 molar ratio), 40 mM, in the absence and presence of 10 mol% artepillin C, at 30°C.

	N_UV_ (%)	d_B_ (Å)	d-spacing (Å)
DOPC/DPPC/Col	52	50.2±0.4	65.8±0.1
DOPC/DPPC/Col + Artepillin C	87	48.0±1.0	71.4±0.5

Data are reported as mean±SD. d_B_: thickness of the lipid bilayer; N_UV_: percentage of unilamellar vesicles; d-spacing: total sum of thickness bilayer (d_B_) plus space between bilayers into the multilamellar vesicles.

### Confocal fluorescence microscopy measurements

Different from the information obtained with the two previous (bulk) techniques at equilibrium, confocal fluorescence microscopy experiments using GUVs allowed us to get spatiotemporal information during the artepillin C/membrane interaction. These two types of information were somehow complementary and helped to obtain a more complete picture of the phenomenon under study.

#### DiIC18 images

**Figure 3.**
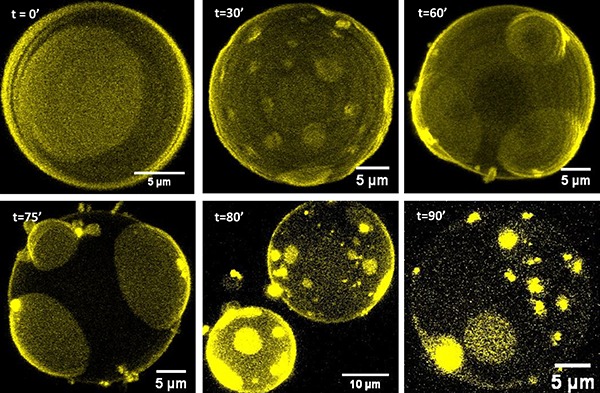
3D projection at Z-plan of giant unilamellar vesicles formed of DOPC/DPPC/cholesterol (23:47:30 molar ratio), labelled with 0.5 mol% of DiIC_18_, obtained by fluorescence confocal microscopy. The images were acquired at different times after addition of artepillin C and analyzed up to 90 min, at 25°C.

Figure 3 shows the time evolution of a three-dimensional projection of a GUV in suspension under the addition of the bioactive compound. Higher and lower fluorescence intensity regions are related to liquid-disordered and liquid-ordered phases, respectively, as reported by Scherfeld et al., 2003 (21). The idea behind this experiment was to test the effect of different amounts of artepillin C on the membranes. The upper left panel of Figure 3 (t=0’), represents the GUV in the suspension in the absence of artepillin C. Sixty minutes after addition of 40 µM of artepillin C, morphological changes were not observed in the GUVs (not shown). In contrast, 30 min after addition of 120 µM of artepillin C, pronounced morphological changes were observed in the GUVs, resulting in the redistribution of l_d_ domains, as shown by the upper middle panel of Figure 3 (t=30'). After 60 min (t=60’ – upper right panel of Figure 3), curvature effects in the vesicles were observed, causing budding of the membrane domains. Interestingly, upon addition of 40 µM more of artepillin C into the suspension at this stage, which corresponds to a total of 160 µM of the bioactive compound, we observed that the liquid-ordered region lost its fluorescence intensity and specific regions with high intensity were concomitantly formed within the liquid-disordered domains, as shown in bottom-left panel of Figure 3, acquired at 75 min (t=75’). If a total of 200 µM of artepillin C is reached (by adding another 40 µM of artepillin C to the sample), both l_o_ and l_d_ regions were affected by the decrease in the probe fluorescence intensity, followed by the creation of highly fluorescent spots in the bilayer (always associated with l_d_ areas), as shown by bottom middle and right panels of Figure 3, at 80 and 90 min (t=80’ and t=90’).

When 200 µM of artepillin C were added at once into the suspension, all effects described above were observed within 14 min, showing that the morphologic changes were dependent on the artepillin C concentration (see Supplementary Figure S2). Formation of fluorescent spots may correspond to a final destabilization of the L_d_ regions, initially showing curvature stress effects caused by artepillin C (budding effect, Figure 3, 60’), when artepillin C reached a given threshold in the membrane. This is supported by the appearance of additional lipid structures in the axial plane of the membrane that were connected (or associated) to the bilayer (Figure 3, t=80’ and t=90’). Interestingly, the results obtained are in agreement with results reported previously, indicating that such effects are mainly caused by the neutral state of artepillin C (23). Although the results described in this section showed several morphological effects on the vesicles caused by artepillin C, further information about the membrane phase state can be obtained with laurdan-GP, as we will present in the next section. The results showed that the effects of artepillin C was independent of curvature radius, once changes in lipid packing were confirmed in both LUVs and GUVs.

#### Laurdan-GP images

**Figure 4.**
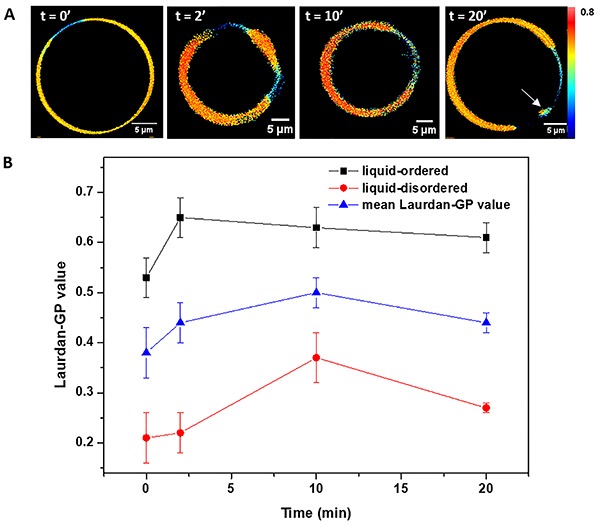
**A**, Representative generalized polarization (GP) images of giant unilamellar vesicles (GUVs) formed of DOPC/DPPC/cholesterol (23:47:30 molar ratio), acquired at different times after addition of 100 µM of artepillin C into lipid suspension, at 25°C. The white arrow (t = 20’) indicates a membrane protrusion occurring in the liquid-disordered region of the membrane. **B**, Laurdan-GP values versus time from regions of interest (ROIs) corresponding to liquid-ordered (black squares) and liquid-disordered (red circles) phases. The blue triangles correspond to GP values obtained from the whole GUV image, i.e., without ROI analysis.

Laurdan-GP image analyses are presented in Figure 4A. The data obtained in the absence of artepillin C was in agreement with the coexistence of L_o_ and L_d_ phases corresponding, respectively, to high and low GP regions (25). No longer than 2 min after exposure to artepillin C, either at 100 or 200 µM, the laurdan-GP calculated from both L_o_ and L_d_ phases (also for the whole vesicle) slightly increased. This result suggests a slight dehydration of both areas caused by artepillin C (Figure 4B). In addition, fluorescent spots as shown from GUVs stained with DiIC_18_ were also observed into L_d_ domains after addition of artepillin C (Figure 4A t=20 min and Supplementary Figure S3). This observation confirmed that this feature can be attributed to small vesicles (or tubes) arising from these areas.

## Discussion

This study has shown alterations in the structural properties and thermal behavior of model bilayers upon interaction with artepillin C in both neutral and deprotonated states. Specifically, when experiments at equilibrium conditions were performed, we noticed that artepillin C produced different effects in the target membrane. For example, upon the addition of 10 mol% of the bioactive compound, a broadening and a shift of T_m_ towards higher temperatures with a concomitant decrease in lipid cooperativity was observed. A previous study showed similar broadening effects in the transition temperature of DMPC vesicles caused by artepillin C, however, T_m_ for that system shifted to lower temperatures. Likewise, by performing DSC experiments for lipid vesicles formed only for DPPC (in the absence of cholesterol), similar effects as reported previously for DMPC system (13) could be seen (Supplementary Figure S4): the higher the concentration of artepillin C, the more broadening and a greater left shift of Tm occurred for the system. In this study, we show results for a temperature range that resulted mainly from the presence of cholesterol embedded in DPPC-enriched domains (L_o_ domains), and the effects observed were different compared to the effects of artepillin C with lipid vesicles formed purely of DPPC. Alves et al. (40) reported the effects for antipsychotic drugs by means of calorimetric studies and they found that the presence of cholesterol embedded to DPPC lipids alters the effects of some drugs compared with purely DPPC system due to their favorable affinity for cholesterol, reflecting an increase in the T_m_ and ΔH. The tendency of artepillin C to increase the T_m_ according to the increase in its concentration may represent a similar affinity for cholesterol as shown by Alves et al. (40). However, the increase in the cooperativity units for higher artepillin C concentration led us to conclude that, despite affinity for cholesterol, artepillin C remained affecting the ordering of DPPC lipids.

It is important to notice that the average thickness (*d*_b_) of the lipid bilayer calculated by means of SAXS experiments was in agreement with the disordering effects pointed out by the calculated CU values. We have found that artepillin C decreased lipid bilayer thickness about 4% for L_o_-L_d_ lipid system. The same thinning effect was previously observed for DMPC vesicles measured in fluid phase (13) and, interestingly, by performing SAXS experiments with lipid bilayer in gel phase (either by using DMPC or DPPC), we found that artepillin C decreased the thickness of the vesicles in a range of 3–4% (data not shown). These features are related to the effects of the neutral species of artepillin C in the lipid membranes, in agreement with the results reported in the literature: in an acidic environment, in the presence of 100% of neutral species of artepillin C the model membrane becomes more fluid, which is less evident for the system displaying 100% of deprotonated species of artepillin C (23). By taking into account the *N_UV_* parameter, we could confirm that artepillin C in its deprotonated state interacted preferentially with polar head group regions, causing an interbilayer electrostatic repulsion and the spontaneous formation of unilamellar vesicles in the lipid suspension, which is also in agreement with previous reports for DMPC and DPPC (13,23).

Using fluorescence microscopy, it was possible to obtain temporal information on the GUV’s morphology upon interaction with artepillin C. For example, redistribution of l_d_ phase domains followed by budding on these membrane regions, which finally destabilized the membrane creating lipid structures connected to the bilayer, were observed in the scale of tens of min. Such results are related to the effects found previously for the neutral state of artepillin C, suggesting that, besides fluidity, artepillin C affects the curvature stress of GUVs (23). This effect was concentration-dependent and, according with the laurdan-GP data (which is sensitive to the amount and dynamics of water molecules existing at the glycerol backbone region of the lipid interface) was accompanied by a slight dehydration of the membrane interface (for both l_o_ and l_d_ phases), mostly due to the presence of deprotonated species in this region. This result was in line with the information obtained with the SAXS data suggesting that artepillin C is located at the membrane surface, i.e., artepillin C may displace water and affect the extent of water relaxation at the membrane interface.

Considering the artepillin C trajectory from the bulk to the lipid surface of DMPC according to a previous study (13), its contact with water decreases over time even after the first contact with the lipid environment, reaching approximately only 5% at the final contact map obtained by simulation. It reinforces the idea that dehydration takes place in the polar head group due to the presence of artepillin C, which could be related to laurdan-GP values calculated by means of microscopy experiments.

In conclusion, the effects of artepillin C on the physical properties of a model membrane mimicking liquid immiscibility provided some clues about its potential biological action, since those lipid structures may play an important role in many biological processes, such as protein trafficking and signaling processes.

## Supplementary material

Click here to view [pdf].
